# Supplementary material for: Detecting horizontal gene transfer among microbiota: an innovative pipeline for identifying co-shared genes within the mobilome through advanced comparative analysis
Source: Microbiol Spectr. 2023 Dec 15;12(1):e01964-23. doi: 10.1128/spectrum.01964-23 (PMC10782964; doi:10.1128/spectrum.01964-23)
Supplement: Supplementary file S1 — Parameter settings on the detection of HGT events using different computational tools. [file spectrum.01964-23-s0001.docx]

**File S1. Detection of HGT events using state-of-art tools: parameter settings**

**DHGT-ComAnalysis** (pipeline presented in this study):

**input sequences:** genome sequences from the dataset, alternatively annotated genomes

Prokka v1.14.5: default settings with *--addgene --locustag strain_name --gcode 11* *--careful* options

UBCG_v3: default settings

iTOL v6: taxonomy metadata file – upon request

dRep v3.4.0: *dRep compare* with *--S_algorithm ANImf --nc 0.9* options

To identify non-redundant pangenome (NRPG) of genomospecies: *cat* genomes within the same genomospecies determined by dREP (≥ 0.95 id) and apply CD-HIT with the settings as for the identification of nearly identical sequences.

To identify nearly identical sequences *cat* all NRPGs of all genomospecies in the dataset and apply CD-HIT v4.7: *cd-hit-est* with *-c 0.99 -s 0.99 -M 0 -d 0 -r 0* settings

For genus and family definition, 16S rDNA sequences were extracted from Prokka annotation and CD-HIT with *-c 0.945* and *0.92* settings were applied, respectively. The NPRG of each genus (family) will be applied accordingly.

eggNOG-mapper v2.1.9: default settings, db: eggNOG 5, input: protein sequences from Prokka

**output results:** list of nearly identical sequences; using *cd-hit-est-id* with the above mentioned settings, nearly identical genes are backtracked to find orthologous genes in isolates, genomospecies, genera etc. HGT interactions between different genera are the foundation for the network analysis.

**MetaCHIP v1.10.13**

**Input sequences:** genome sequences from the dataset

taxonomic db: GTDB_Tk release 214

MetaCHIP PI and MetaCHIP BP with *-r pcofg* option

**Output results:** HGT interactions between different genera, families, etc.; list of donor and recipient gene sequences; chord diagrams of HGT network

**ShadowCaster 0.9.2**

**Input sequences**: protein-coding DNA sequences (analyzed genome *Phocaeicola sp900066445* 1_COKtk)

*query proteome: amino acid sequences of all proteins from the genomes within the dataset* (annotated by Prokka)

*nuSVM = 0.4*

**Output results**: list of HGT candidates within the genome

**Alien_hunter 1.7**

**Input sequences**: genome sequences from the dataset

default settings

**Output results**: .embl files containing coordinates of predicted HGT sequences

**HGTector2**

**Input sequences**: amino acid sequences of all proteins (analyzed genome *Phocaeicola sp900066445* 1_COKtk)

db: all protein sequences of NCBI RefSeq genomes of bacteria, archaea, fungi and protozoa default parameters with *--donor-name* option

**Output results**: list of HGT genes and putative donor organism
