# Supplementary material for: Detecting horizontal gene transfer among microbiota: an innovative pipeline for identifying co-shared genes within the mobilome through advanced comparative analysis
Source: Microbiol Spectr. 2023 Dec 15;12(1):e01964-23. doi: 10.1128/spectrum.01964-23 (PMC10782964; doi:10.1128/spectrum.01964-23)
Supplement: List of Supplementary Material — Legends for all supplementary files. [file spectrum.01964-23-s0005.docx]

**SUPPLEMENTARY MATERIAL**

**Supplementary Figure S1:** Phylogenetic tree inferred by the RAxML using gene sequences coding for 16S rDNA genes. Families within the phylum *Bacteroidetes* are in shades of purple, within the phylum *Firmicutes* are in green, within the phylum *Proteobacteria* are in blue, and within the phylum *Actinobacteria* are in yellow, within the phylum *Fusobacteria* in orange, within the phylum *Verrucomicrobia* are in beige, within the phylum *Elusimicrobia* are in golden brown, and within the phylum *Synergistetes* are in pink. Bar, 1 substitution per position.

**Supplementary Figure S2**. Detailed resolution heatmap showing the abundance of genes co-shared by two different genomospecies backtracked to individual genomes with the emphasis on the visualization of low-abundant HGT genes. Mind that we used logarithmic-scale color bar. Individual genome (coloring as in Figure 1) were ordered according the UBCG tree.

**Supplementary Figure S3**. Network visualization of genes co-shared by different isolates and genomospecies with the emphasis on the origin of isolates. Genomes of isolates presented as nodes; the same genomospecies are clustered into circle together. Edges represents the number of mobile elements transferred using edge transparency (none or white color represents zero co-transferred genes, black represents the maximum number of transferred genes, it was 548 between SAMN15872594 and SAMN15872602). Porcine isolates are marked in pink, whereas chicken isolates are in yellow.

**Supplementary Table S1**. List of analyzed samples.

**Supplementary Table S2**. List of identified HGT genes.

**Supplementary file S1.** Parameter settings on the detection of HGT events using different computational tools
